# Prenatal Exposure to Cadmium, Placental Permeability and Birth Outcomes in Coastal Populations of South Africa

**DOI:** 10.1371/journal.pone.0142455

**Published:** 2015-11-06

**Authors:** Halina B. Röllin, Tahira Kootbodien, Kalavati Channa, Jon Ø. Odland

**Affiliations:** 1 School of Health Systems and Public Health, Faculty of Health Sciences, University of Pretoria, Pretoria, South Africa; 2 Environment and Health Research Unit, Medical Research Council, Johannesburg, South Africa; 3 Lancet Laboratories, Department of Analytical Chemistry, Johannesburg, South Africa; 4 Institute of Community Medicine, University of Tromsø, Tromsø, Norway; University of Missouri, UNITED STATES

## Abstract

**Background:**

The impact of prenatal exposure to cadmium (Cd) on birth outcomes is an area of concern. This study aimed to assess an impact of prenatal Cd exposure on birth outcomes in distinct coastal populations of South Africa.

**Methods:**

Cadmium was measured in maternal blood (CdB) (n = 641), cord blood and in maternal urine (n = 317). This investigation assessed the associations between CdB (non-transformed) and birth outcomes across the 25^th^, 50th, and 75th percentile for birth weight, birth length and head circumference, to test for a linear trend. Associations between natural log-transformed maternal CdB, size at birth and other factors were further evaluated using linear mixed-effects modelling with random intercepts.

**Results:**

The average gestational age in the total sample was 38 weeks; 47% of neonates were female, average birth weight was 3065 g and 11% were of low birth weight (< 2500 g). The geometric mean (GM) of the maternal CdB level was 0.25 μg/L (n = 641; 95% CI, 0.23–0.27). The cord blood Cd level was 0.27 μg/L (n = 317; 95% CI, 0.26–0.29) and urine (creatinine-corrected) Cd level was 0.27 μg/L (n = 318; 95% CI, 0.24–0.29). The CdB cord:maternal ratio in the sub-cohort was 1, suggesting that the placenta offers no protective mechanism to the foetus. An inverse association was found between CdB and the lower birth weight percentile in female neonates only (*β* = - 0.13, *p* = 0.047). Mothers who reported eating vine vegetables daily had lower levels of CdB (*β* = - 0.55, *p* = 0.025). Maternal smoking was associated with an elevation in natural log-transformed CdB levels in both male and female cohorts.

**Discussion:**

Significant inverse associations between prenatal Cd exposure and birth anthropometry were found in female neonates but not in male neonates, suggesting potential sex differences in the toxico-kinetics and toxico-dynamics of Cd.

## Introduction

The impact of prenatal exposure to cadmium (Cd) on birth outcomes and childhood development is an area of concern and a subject of vigorous research, mainly in the northern hemisphere, while there continues to be a dearth of data from the southern hemisphere.

Cadmium is a highly toxic pollutant present in the living environment that is detrimental to human health. The main source of Cd to humans is food, exposure to tobacco smoke, industrial activities and contaminated drugs and dietary supplements [1–3]. Cd is considered to be a major pollutant in the northern hemisphere [4, 5] where it has been shown to accumulate in lichens and other plants that are consumed by herbivorous mammals and consequently ingested by the indigenous population consuming traditional foods, mainly wild game [6–8].

Currently, it is understood that the exposure to low doses of Cd from all sources may produce long-term health effects in humans [9]. Worldwide, exposure to Cd in the general population is actually rising, except in areas where contamination was once high and remediation procedures were applied [10].

Cadmium is nephrotoxic and carcinogenic in humans and has been shown to be embryotoxic and teratogenic in animal models [11]. The main route of Cd uptake in humans is by inhalation and ingestion, with dermal absorption considered to be negligible. It is estimated that, depending on the particle size, about 10–50% of inhaled Cd is absorbed by humans. If ingested, about 5–10% of Cd is absorbed, depending on the individual’s status of essential elements such as iron, calcium and zinc [12, 13]. After uptake, Cd in blood binds to albumin and a sulfhydryl group containing the protein metallothionein (MT) causing tubular damage that is proportionate to the quantity of Cd not bound to the MT [14]. It has been shown that diabetics, even at low exposure to Cd, are more susceptible to renal tubular damage, when compared to healthy populations [15]. Furthermore, Cd may also cause an impairment of vitamin D metabolism in the kidney, impacting on bone and leading to osteomalacia [16, 17].

It is now well understood that health risks from exposure to environmental pollutants are age and life stage related, and that prenatal exposures may have a direct impact not only on birth outcomes and early childhood development but also on health at all life stages of the individual [18]. It has been shown in animal experiments that prenatal exposure to Cd is a critical window for its toxic effects. Furthermore, animal studies indicate that exposure to Cd may have endocrine effects by mimicking the *in vivo* effects of oestrogen in the uterus, and mammary glands in rats, resulting in earlier onset of puberty, as well as affecting post-natal immune function. Some of these detrimental effects are already evident at low-level environmental exposures to Cd [19–21].

During the prenatal stage, Cd has not only the ability to accumulate in the placenta, but also the ability to permeate the placenta and interfere with the transport of various micronutrients to the foetus [22–24]. Recent research suggests that prenatal exposure to Cd, known to be an epigenic modifier, may also influence long-term childhood development [25]; in addition, prenatal exposure to even low levels of Cd may have a detrimental effects on birth outcomes [26–28].

The aim of this study was to investigate prenatal exposure to Cd from all sources, and its possible impact on birth outcomes in South African coastal populations, by measuring the maternal blood levels of Cd in a cohort of 641 women at the time of delivery. Furthermore, a sub-cohort of 317 women, from whom paired cord blood and maternal urine samples were collected, was assessed for a possible correlation between Cd levels in three biological parameters, viz. maternal blood, maternal urine and cord blood, which may be directly related to Cd exposure.

This study is part of on-going multidisciplinary, multi-institutional research collaboration between South Africa and Norway—the Arctic Monitoring and Assessment Programme (AMAP)—that evaluates exposures and effects of persistent toxic substances (PTS) on reproductive health and birth outcomes in populations of the northern and southern hemispheres.

## Materials and Methods

### Study sites

**Fig 1:**
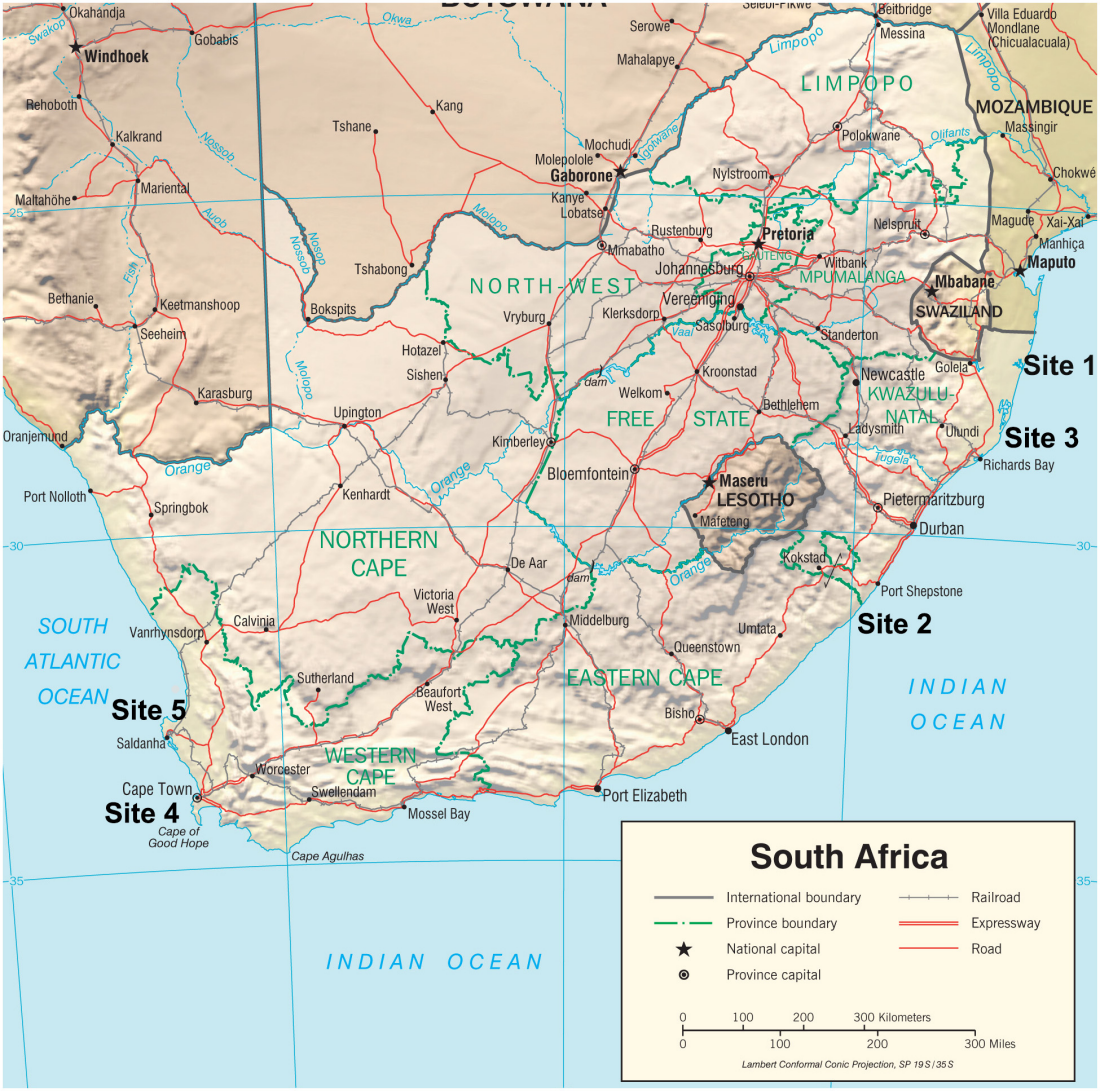
Study sites: sites 1, 2, 3—Indian Ocean; sites 4 and 5—Atlantic Ocean. Figure is identical but sites locations were added, and is therefore for representative purposes only. https://www.cia.gov/library/publications/resources/cia-maps-publications/South%20Africa.html.

A total of five sites were included in the study: three sites were situated along the Indian Ocean coast of the KwaZulu Natal (KZN) Province, and two sites were situated along the Atlantic Ocean coast of the Western Cape Province of South Africa (Fig 1).

All three sites along the Indian Ocean coast can be defined as rural with varying degrees of agricultural activities. However, their geographical location may predispose them to environmental pollution emanating from mining and industrial activities (site 1); aluminium smelting, mining, coal terminals and ports (site 2); and small industrial activities (site 3). Along the Atlantic Ocean coast, one study site is the urban city of Cape Town, which is surrounded by commercial, industrial and port activities (site 4); and the second site is considered to be rural (site 5), where commercial agriculture and fishing activities dominate.

### Participants

Potential study candidates were recruited by a health worker on duty, from women who were admitted for delivery at the local maternity sections of public hospitals. A trained research assistant briefly explained the objectives of the study and distributed a detailed information sheet about the project. All women who agreed to participate signed an informed consent form and agreed to donate blood before delivery. Participants agreed to answer a socio-demographic questionnaire which also included the topics of diet, lifestyle, and health status. Participants also consented to access and use of hospital birth outcome data for research purposes. The socio-demographic questionnaire was not specifically designed for Cd exposure, but for exposure to environmental pollutants in general. The dietary part of the questionnaire recorded the intake frequency of various basic foods during pregnancy. After delivery, records from hospital files were extracted, including maternal and neonate characteristics such as weight, length and head circumference, gestational age, as well as any birth complications, if any. In total, 650 delivering women participated in the study, of which 641 women, who delivered singleton infants of gestation of more than 20 weeks, were included. Due to financial constraints, the collection of additional samples (pre-partum urine samples and post-partum cord blood samples) was limited to women residing in Sites 1, 2 and 3 (n = 317).

### Sampling procedure

From each woman, a volume of 10 ml of venous blood was collected before delivery into BD Vacutainer tube (10 ml capacity and containing EDTA). Participants from the sub-cohort (n = 317) also donated 30 ml of urine before delivery, and 10 ml of umbilical cord blood post-partum.

### Sample processing and analytical procedure

The analyses for Cd content in whole blood and urine were performed using an Inductively Coupled Plasma-Mass Spectrometer (ICP-MS) instrument (Agilent 7500ce ICP-MS with an Octopole Reaction System). Contamination-free vessels and procedures were used throughout, and validation of results was accomplished by including certified standards, as well as certified references quality controls, in the analyses.

Briefly, the whole blood samples (0.5ml volumes) were digested with 1 ml nitric acid (Fluka, Trace Select Ultra for trace analysis) at 90°C for 2 hours. After cooling, the internal standard was added and samples were diluted with water to a final volume of 7 ml. Cd was measured in ‘no gas’ acquisition mode with ^115^In and ^197^Au as internal standards. Aliquots of each sample were analysed in triplicate. The detection limits (three times the standard deviation of all blank samples) for Cd was 0.03μg/L. For quality assurance, two certified reference controls, viz. Seronorm ^™^Trace Elements (Sero LTD., Billingstad, Norway) in whole blood (Levels 1 and 2), were analysed with every analytical run, at intervals between every 10 samples. Urine samples (1 ml volumes) were digested with 0.1 ml of 65% ultrapure nitric acid ((Fluka, Trace Select Ultra for trace analysis). An internal standard solution containing ^72^Ge, ^115^In and ^197^Au was added (50 μl) to all samples, reagent blanks, reference controls and calibration standards and made up to 5 ml (5 times sample dilution). Urinary Cd (CdU) levels were measured in ‘no gas’ acquisition mode and percentage recovery, when using certified standards (Seronorm Urine Blank and Lyphchek Level 1&2), was 108.8% and 111.5% for level 1 and 2, respectively.

The sample analyses were conducted by the Johannesburg National Institute for Occupational Health (NIOH) laboratory, which participates in the Wadsworth Center—New York State Department of Health Proficiency testing scheme for whole blood and urine. The results obtained are consistently accepted with no indication of bias.

### Covariates

Covariate information was obtained during the questionnaire-based interview and from medical records. Maternal weight and height were recorded at the hospital on admission. From the medical records, the following newborn characteristics were retrieved: birth weight (grams), birth length (cm), head circumference (cm) and gestational age (weeks). Preterm labour was defined as mothers giving birth at less than 37 weeks gestational age. Education was categorised as no education, completed primary school, completed secondary school and any level of tertiary education reached. Maternal tobacco smoking was defined as ever or never. Exposure to environmental tobacco smoke (ETS) was defined as exposure to tobacco smoke from smoking by others in the household. A binary classification was used for exposure to indoor smoke from the burning of fossil fuel (wood and coal) for the purpose of heating or cooking, separating women into those exposed to fossil fuel and those not exposed (for example, electricity). Dietary questions relating to vine, root and leafy vegetable intake were assessed and classified as daily, at least once a week and seldom.

### Statistical analyses

The statistical analyses were performed using STATA (StataCorp, 2013. Stata Statistical Software: Release 13. College Station, TX: StataCorp LP). The distribution of Cd levels in maternal blood and urine and in cord blood, were skewed and were log transformed. Bivariate analyses between Cd exposure, sizes at birth and covariates were evaluated by Spearman’s correlation coefficient. The non-parametric Wilcoxon rank-sum (Mann-Whitney) and Kruskal-Wallis rank tests were used where appropriate, to make categorical comparisons of the maternal CdB distribution (n = 641) and demographic, dietary, and environmental characteristics. The 25th percentile (lowest quintile), 50th percentile (median quintile), and 75th percentile (highest quintile) were assessed for birth outcomes (birth weight, birth length and head circumference). Furthermore, associations between CdB (non-transformed) and size at birth across the quintiles were assessed using a non-parametric test (*nptrend* in Stata) to test for linear trend. Linear mixed-effect models with random intercepts were used to identify significant predictors (*p* < 0.1) of natural logarithm—transformed blood Cd levels and to estimate the amount of variability in measured levels explained by the model. Potential explanatory variables were identified from questionnaire data that were associated with blood Cd levels with a *p* < 0.2 during univariate analyses: vegetable intake, smoking history, burning of fossil fuel, environmental tobacco smoke exposure, gestational age, parity, size at birth (birth weight, birth length and head circumference), and gender of the newborn. Model fit was compared using log likelihood and Akaike Information Criterion (AIC) statistics.

### Ethical considerations

Ethics approval for the study was obtained from the Human Research Ethics Committee of the University of Witwatersrand in Johannesburg (Protocol no. M10742), and from the relevant provincial Departments of Health. The sites along the Indian Ocean coast (sites 1 to 3) fall under the Provincial Department of Health of KwaZulu Natal, which issued ethical consent for each site and requested the CEOs of the respective hospitals to allow this research to take place. Each CEO confirmed that he/she allowed the research work to proceed. The sites along the Atlantic Ocean coast (sites 4 and 5) fall under the Western Cape Provincial Department of Health; identical procedures were followed in terms of obtaining consent for the study. Confidentiality was maintained by assigning identification numbers to all study participants. During the informed consent process, it was emphasised that participation was voluntary and could be withdrawn at any time.

## Results

### Characteristics of the study population

The background characteristics and Cd concentrations of the mothers are presented in Table 1.

**Table 1:** Characteristics of the study populations (mothers and newborn) and cadmium levels in maternal blood and urine, and in cord blood.

Characteristics	n	Mean ± SD (range) or percent	Median	25^th^-75^th^ percentile
**Maternal**				
Age (years)	637	25.2 ± 6.2 (14–49)	24	15–42
Weight (kg)^b^	607	73.4 ± 15.5 (40–143)	71.6	41–128
Height (cm)	485	157.8 ± 10.1 (59–183)	159	124–176
BMI (kg/m^2^)	476	29.2 ± 6.8 (16.4–68.6)	28.3	17.8–52.3
< 18.5	6	1.3		
≥ 18.5	470	98.7		
Parity	622	1.03 ± 1.27 (0–8)	1	0–6
Primiparous	272	43.7		
Multiparous	350	56.3		
**Tobacco smoking**				
Never smoked	484	74.5		
Ever smoked	166	25.5		
Environmental tobacco smoke exposure (yes/no)	626	40.2/59.8		
Burning of fossil fuel (yes/no)	602	36.6/63.4		
**Newborns**				
Sex (male/female)	609	52.8/47.2		
Gestational age (weeks)	549	38.2 ± 2.2 (24–44)	38	30–43
< 37	72	13.1		
≥ 37	477	86.9		
Weight (g)	618	3065 ± 515.2 (855–5150)	3100	1300–4300
< 2500	66	10.7		
≥ 2500	552	89.3		
Length (cm)	602	49.5 ± 3.5 (31–66)	50	35–58
Head circumference (cm)	604	34.7 ± 2.1 (25–50)	35	28–45
**Cadmium concentrations**				
Blood Cd (μg/L) ^**a**^	641	0.25 (0.23–0.27)	0.26	0.04–3.73
Creatinine-corrected Urine Cd (μg/L) ^**a**^	317	0.29 (0.27–0.31)	0.29	0.18–0.47
Urine Cd (μg/L) ^**a**^	317	0.27 (0.24–0.29)	0.26	0.03–2.05
Cord Cd (μg/L) ^**a**^	317	0.27 (0.26–0.29)	0.28	0.09–0.92

^a^Geometric mean (95% CI),

^b^weight at admission

Maternal age ranged from 14 to 49 years, with the average being 25 years; approximately 44% of the women were primiparous. Their average BMI at admission was 29 kg/m^2^. Approximately 26% of the women reported ever using tobacco, 40% reported exposure to environmental tobacco smoke in the household, and 37% burned fossil fuel for heating or cooking in their homes. Table 1 also shows the descriptive data of the newborns; 47% were female, the average gestational age was 38 weeks (range: 24–44 weeks), and 13% were born prematurely. Birth weight ranged from 855g to 5150g, with an average of 3065g. Male infants (mean, 3119 g) weighed more than females (mean, 3030 g, *p* = 0.04). Additionally, 66 infants (11%) were considered to be of low birth weight.

### Cd levels in the study population

In the total study sample, the geometric mean (GM) of the maternal CdB level was 0.25 μg/L (95% CI, 0.23–0.27) and the 50^th^ percentile was 0.26 μg/L, with an interquartile range of 0.04 μg/L to 3.73 μg/L. The GM of the cord Cd level was 0.27 μg/L (n = 317; 95% CI, 0.26–0.29) and the GM of the CdU level was 0.27 μg/L (n = 318; 95% CI, 0.24–0.29). Among the 317 mothers of the sub-cohort, the GM maternal CdB level was 0.26 μg/L (95% CI, 0.23–0.28). Since the ratio of maternal to cord CdB was found to be 1:1, we deduced that no placental protective effect was demonstrated.

There was no correlation between maternal CdB and cord Cd levels (*rho* = 0.08, *p* = 0.155) or between maternal CdU and cord Cd levels (*rho* = 0.002, *p* = 0.969). A weak association was found between maternal CdB and CdU levels (*rho* = 0.11, *p* = 0.062).

Maternal CdB levels did not differ between multiparous (GM, 0.25 μg/L) and primiparous mothers (GM, 0.24 μg/L; *p* = 0.772). Mothers of infants who were born preterm had a higher GM for CdB levels (0.28 μg/L), compared to the mothers of infants born at term (0.24 μg/L); however this finding was not significant (*p* = 0.337).

The distribution of maternal CdB levels (μg/L) at delivery, by smoking history, ETS exposure and burning of fossil fuel is described in Table 2.

**Table 2:** Distribution of maternal blood cadmium (CdB) levels (μg/L) by smoking history, environmental tobacco smoke (ETS) exposure and burning of fossil fuel.

Variable	n	Geometric mean (95% CI)	Median	25^th^-75^th^ percentile
Tobacco smoking				
Never smoked	484	0.22 (0.20–0.24)	0.22	0.04–2.73
Ever smoked	166	0.36 (0.32–0.41)	0.36	0.05–1.97
Environmental tobacco smoke (ETS) exposure				
No	377	0.22 (0.20–0.24)	0.22	0.03–2.23
Yes	249	0.29 (0.26–0.33)	0.32	0.04–2.51
Burning of fossil fuel				
No	381	0.26 (0.24–0.29)	0.31	0.04–2.23
Yes	221	0.22 (0.19–0.24)	0.21	0.04–1.45

Maternal CdB levels were significantly higher among mothers who reported ever having smoked (GM, 0.36 μg/L vs 0.22 μg/L, Wilcoxon rank-sum, *p* < 0.001) and mothers exposed to ETS in their household (GM, 0.29 μg/L vs 0.22 μg/L, Wilcoxon rank-sum, *p* = 0.001). Interestingly, mothers who burned fossil fuel in their household for heating or cooking were found to have significantly lower maternal CdB levels (GM, 0.21 μg/L vs 0.26 μg/L, Wilcoxon rank-sum, p = 0.003).

Mothers who consumed vine vegetables at least once a day had significantly higher blood Cd levels (GM, 0.28 μg/L) than mothers who seldom consumed vine vegetables (GM, 0.21 μg/L) or consumed vine vegetables once a week (0.19 μg/L; *p* = 0.001). Mothers who seldom ate root vegetables had significantly higher CdB levels (GM, 0.64 μg/L) than mothers who ate root vegetables once a week (GM, 0.23 μg/L) or daily (GM, 0.25 μg/L; *p* = 0.002). Maternal CdB levels did not differ between mothers who seldom ate leafy vegetables (0.36 μg/L) and mothers who ate leafy vegetables at least once a week (GM, 0.23 μg/L) or daily (GM, 0.25 μg/L, *p* = 0.169).

In the bivariate analysis (see Table 3), maternal CdB was negatively correlated with birth weight and maternal height and was weakly correlated with creatinine-corrected CdU.

**Table 3:** Spearman’s rank correlation coefficient (p-value) of associations between exposures, maternal covariates and infant anthropometry measures at birth.

Variable	Birth weight (g)	Birth length (cm)	Head circumference	Maternal blood Cd (μg/L)
Maternal CdB	-0.112 (0.002)*	-0.055 (0.176)	-0.069 (0.089)	-
Urinary Cd (CdU)	-0.002 (0.996)	-0.008 (0.883)	0.079 (0.178)	0.105 (0.062)
Creatinine-corrected CdU	-0.029 (0.608)	-0.038 (0.519)	0.060 (0.306)	0.137 (0.015) *
Cord Cd (CdC)	-0.026 (0.664)	-0.103 (0.074)	0.101 (0.079)	0.088 (0.155)
Age	0.078 (0.052) *	0.053 (0.191)	0.010 (0.803)	-0.020 (0.616)
Parity	0.059 (0.140)	-0.017 (0.688)	-0.048 (0.237)	-0.011 (0.771)
Maternal weight	0.131 (0.002) *	0.111 (0.008) *	0.201 (<0.001) *	-0.008 (0.839)
Maternal height	0.128 (0.006) *	0.072 (0.123)	0.102 (0.029) *	-0.157 (<0.001) *

* Indicates significance (p<0.05)

Maternal CdB was not significantly correlated with maternal age, parity or maternal weight. In addition, birth weight was positively correlated with maternal age, maternal weight and maternal height.

**Fig 2:**
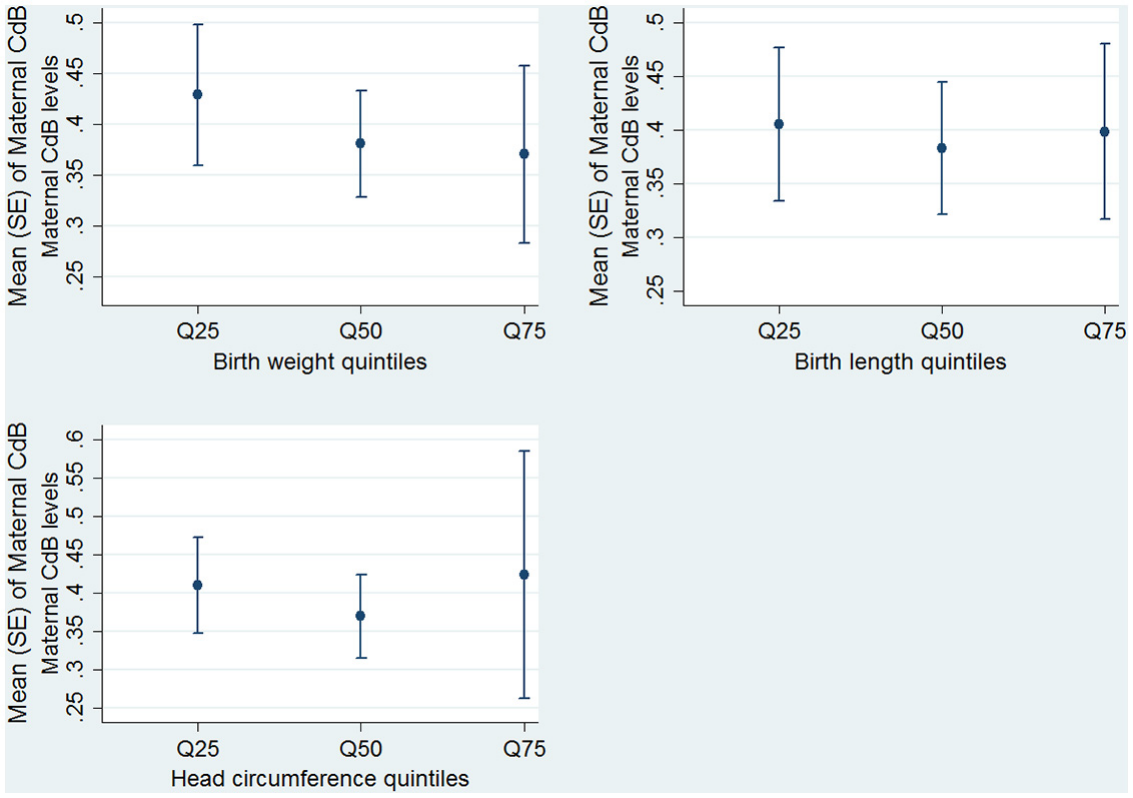
Mean maternal blood cadmium (CdB) levels in each quintile [25th (Q25), 50th (Q50), and 75th (Q75)] of birth weight, birth length and head circumference in the total sample.

In Fig 2, the mean CdB levels are shown for each quintile [25th (Q25), 50th (Q50), and 75th (Q75)] of birth weight, birth length and head circumference in the total sample.

Mean CdB levels were highest at the 25^th^ percentile for birth weight and decreased as birth weight increased (p-value for trend, *p* = 0.001). There was no trend found across birth length (*p*-value for trend, *p* = 0.386) or head circumference quintiles (*p*-value for trend, *p* = 0.192).

**Fig 3:**
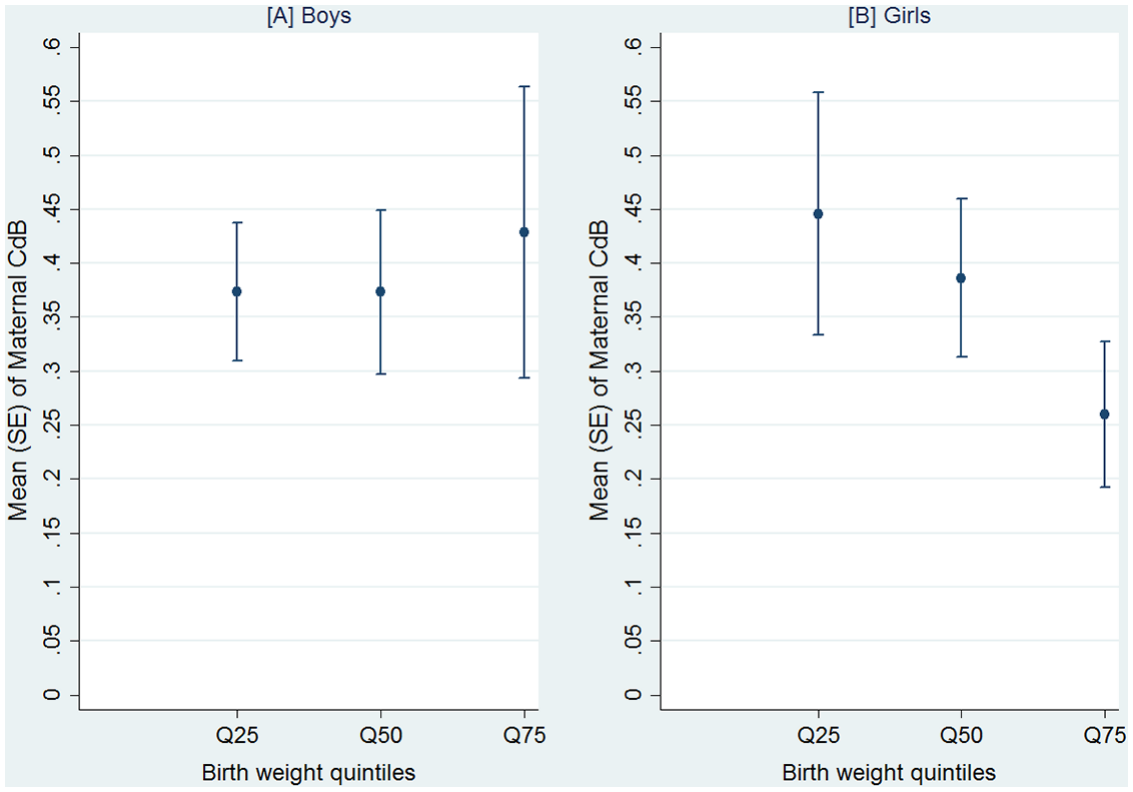
Mean maternal blood cadmium (CdB) levels in each quintile [25th (Q25), 50th (Q50), and 75th (Q75)] of birth weight for [A] boys and [B] girls.

The association between maternal CdB levels and size at birth by gender was also investigated. Fig 3 shows the distribution of CdB levels in each quintile of birth weight by gender.

For female infants, the highest mean maternal CdB levels were reported for the lowest birth weight percentile. Specifically, maternal CdB levels decreased as birth weight increased (*p*-value for trend, *p* < 0.001). No significant trend was shown by birth length (*p*-value for trend, *p* = 0.143) or head circumference (*p*-value for trend, *p* = 108). In male infants, no significant trend was shown by birth weight (*p*-value for trend, *p* = 0.274), birth length (*p*-value for trend, *p* = 0.773) or head circumference (*p*-value for trend, *p* = 0.664).

In the total cohort, after adjusting for maternal weight and ETS exposure, infants with larger head circumference (Q50) were associated with lower natural log transformed maternal CdB levels (*β* = - 0.08, *p* = 0.017, see Table 4).

**Table 4:** Multivariable linear mixed regression analyses of the association between natural log transformed maternal CdB levels and size at birth in total sample and by gender.

	All			Boys (n = 322)			Girls (n = 287)		
Predicted	β (95% CI)	*p*-value	Adjusted *p*-value	β (95% CI)	*p*-value	Adjusted *p*-value	β (95% CI)	*p*-value	Adjusted *p*-value
**Birth weight quintile**									
Q25	Reference			Reference			Reference		
Q50	0.03 (-0.04, 0.10)	0.913	0.478	-0.014 (-0.12, 0.09)	0.998	0.798	0.05 (-0.05, 0.15)	0.775	0.335
Q75	-0.05 (-0.14, 0.03)	0.115	0.216	-0.007 (-0.13, 0.11)	0.885	0.906	-0.13 (-0.25, -0.01)	0.007	0.047
**Birth length quintile**									
Q25	Reference			Reference			Reference		
Q50	0.05 (-0.02, 0.12)	0.399	0.163	0.12 (0.01, 0.22)	0.206	0.030	-0.005 (-0.11, 0.09)	0.856	0.919
Q75	0.02 (-0.06, 0.09)	0.433	0.711	0.08 (-0.04, 0.20)	0.432	0.183	-0.062 (-0.18, 0.06)	0.201	0.316
**Head circumference**									
Q25	Reference			Reference			Reference		
Q50	-0.08 (-0.16,-0.01)	0.117	0.017	-0.09 (-0.19, 0.01)	0.279	0.070	-0.06 (-0.15, 0.04)	0.141	0.236
Q75	-0.09 (-0.20, 0.08)	0.044	0.071	-0.05 (-0.19, 0.08)	0.433	0.455	-0.15 (-0.32, 0.01)	0.023	0.066
**Vine vegetable intake**									
Seldom	Reference			Reference			Reference		
Once a week	-0.42 (-0.78, -0.04)	0.047	0.029	-0.33 (-0.89, 0.22)	0.073	0.239	-0.33 (-0.84, 0.17)	0.158	0.195
Daily	-0.57 (-0.93, -0.20)	0.004	0.003	-0.41 (-0.97, 0.14)	0.023	0.149	-0.55 (-1.04, -0.07)	0.056	0.025
**Root vegetable intake**									
Seldom	Reference			Reference			Reference		
Once a week	-0.42 (-0.79, -0.04)	0.872	0.331	0.09 (-0.14, 0.32)	0.765	0.466	0.05 (-0.23, 0.32)	0.573	0.714
Daily	-0.57 (-0.93, -0.19)	0.861	0.044	0.12 (-0.11, 0.35)	0.758	0.303	0.22 (-0.05, 0.49)	0.301	0.109
**Tobacco smoke**	0.14 (0.06, 0.22)	< 0.001	< 0.001	0.13 (0.03, 0.24)	< 0.001	0.013	0.19 (0.08, 0.30)	0.008	0.001
**ETS exposure**	-0.04 (-0.11, 0.02)	0.002	0.176	-0.07 (-0.16, 0.01)	0.013	0.093	-0.02 (-0.12, 0.07)	0.107	0.628
	*Wald χ^2^ test (14) = 50*.*27*, *p < 0*.*001*			*Wald χ^2^ test (13) = 20*.*18*, *p = 0*.*04*			*Wald χ^2^ test (13) = 52*.*82*, *p <0*.*001*		

Mothers who consumed vine (*β* = - 0.57, *p* = 0.003) and root vegetables (*β* = - 0.57, *p* = 0.044) daily were associated with lower natural log transformed maternal CdB levels. As expected, mothers who reported ever having smoked were associated with higher natural log transformed maternal CdB levels (*β* = 0.14, *p* < 0.001).

In the stratified analyses, the inverse association between birth weight and maternal CdB was shown only for female newborns (*β* = - 0.13, *p* = 0.047). Also, mothers who consumed vine vegetables daily had decreased CdB levels, thereby demonstrating a potential protective effect (*β* = - 0.55, *p* = 0.025). In both male and female newborn cohorts, maternal smoking was associated with an elevation in natural log transformed maternal CdB levels.

## Discussion

The current study has investigated the effects of prenatal exposure to Cd on the size of neonates at birth, in women living along coastal communities in South Africa. To the best of the authors’ knowledge, this is the first study to date that has examined this association in a large cohort of South African women. In the total study population, maternal CdB levels were found to be similar to those reported in the USA study—National Health and Nutrition Examination Survey (NHANES) [29] and the North Norwegian Mother and Child Study [29–31]. Overall there was no differences in CdB levels between the total and the sub-cohort populations nor between multiparous and primiparous mothers. Mothers of infants who were born preterm had higher GM of Cd levels, when compared to mothers whose infants were born at term, but this association was not statistically significant. Maternal age and education had no effect on Cd levels in this study.

When evaluating placental permeability with regard to CdB, the placenta did not appear to function as an efficient barrier against Cd passage, as previously reported [24, 32–34]. However it should be noted that in the pilot study performed in different geographical regions within South Africa, cord blood Cd levels were found to be much lower than the corresponding maternal levels, although not statistically significant [35].

The current study did not find correlations between maternal CdB levels and cord CdB levels, and a weak association was found between maternal CdB levels and creatinine-corrected urine CdU levels. In the majority of the studies performed in the general and occupationally exposed population, as well as in some studies on pregnant women, the CdU level has been used as the “biomarker” of choice for Cd exposure. The findings from this study suggest that more research is required to identify the most suitable biomarker for Cd exposure in humans [28]. The urine Cd levels in this study were found to be very similar to those reported in the studies from USA and Europe [29–31], but much lower than the CdU levels reported in rural Bangladesh pregnant women who did not smoke [28]. Despite emerging evidence of cadmium associated detrimental birth outcomes, there are no consensus on levels of concern for pregnant women or general population.

In line with the latest research on gender related differences in exposure and the resulting health effects caused by metals, the association between maternal Cd concentration and size of neonates at birth, by gender, was investigated [36]. Among female neonates, the highest mean maternal CdB levels were reported for the lowest birth weight percentile. No significant trend was observed for birth length or head circumference. In the male neonates, no significant trend was evident for any of the three birth outcomes. These results add to the accumulating literature on sex-specific differences, in terms of associations between maternal CdB and foetal growth [37].

Protective effects of diet were found for all infants of mothers who consumed vine and root vegetables daily, suggesting a low Cd content in agricultural soil or an inability of these vegetables to absorb Cd in the coastal geographical area under investigation, as most of the women reported growing their own vegetables.

Although, our study was performed on a large cohort of women, there are number of limitations which need to be considered. The first is a recall bias in dietary intake. Secondly, due to financial constraints, the collection of additional samples (pre-partum urine samples and post-partum cord blood samples) were limited to women residing along Indian Ocean coast only, which might have introduced some bias. It also appears that women in the sites along the Indian Ocean coast may have under reported their smoking habits, and since cotinine was not measured it is impossible to verify the answers given in their socio-demographic questionnaires.

## Conclusions

The investigation was performed on a fairly large sample size of 641 women who resided in five geographically distinct coastal sites of South Africa. The main strength of this study is the measurement of Cd levels in three biological parameters, viz. in the maternal blood and in maternal urine and cord blood samples, in the case of the sub-cohort of women. A significant inverse association was found between prenatal Cd exposure and birth weight in female neonates, but none in male infants, confirming the potential for sex response differences to Cd exposure. Of concern is the fact that low birth weight was evident at relatively low Cd concentrations in all three biological parameters, especially as it has been suggested that low-level environmental Cd exposure in children may be associated with adverse neurodevelopmental outcomes.

Based on these findings, the authors recommend that Cd levels be more thoroughly investigated across pregnancy and post-partum, so as to identify prevention methods for foetal Cd exposure, from a public health perspective.

## Supporting Information

S1 Results (DOCX)
